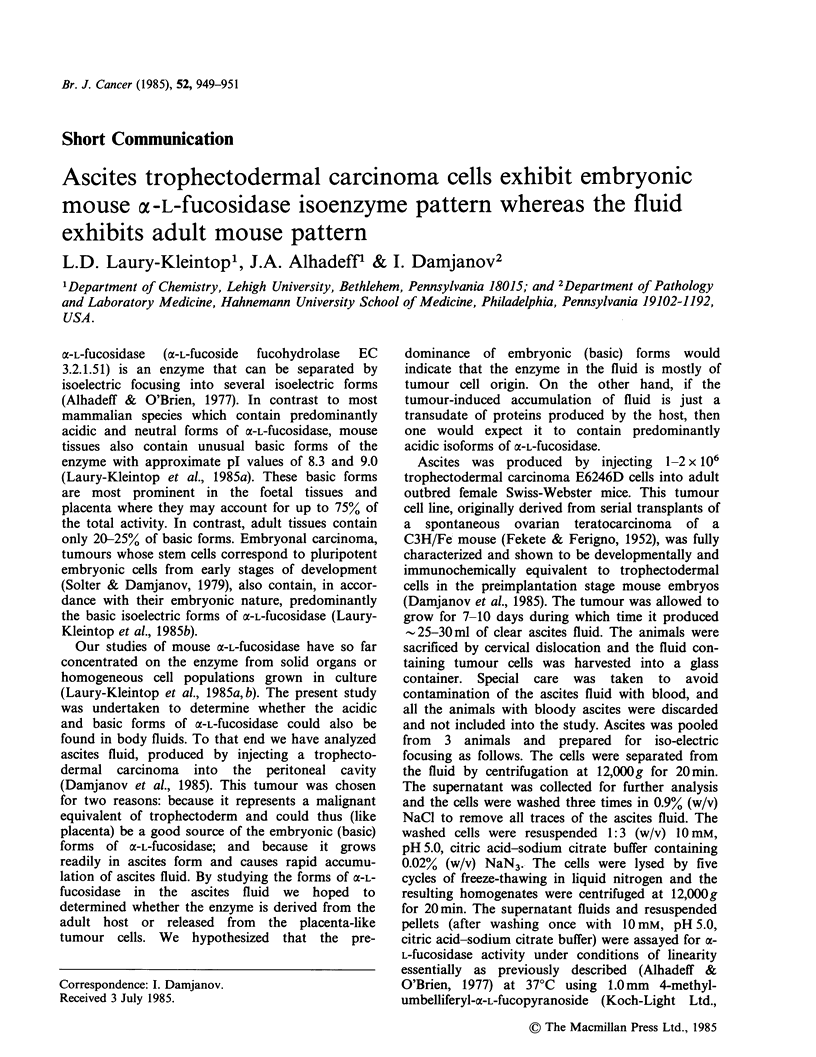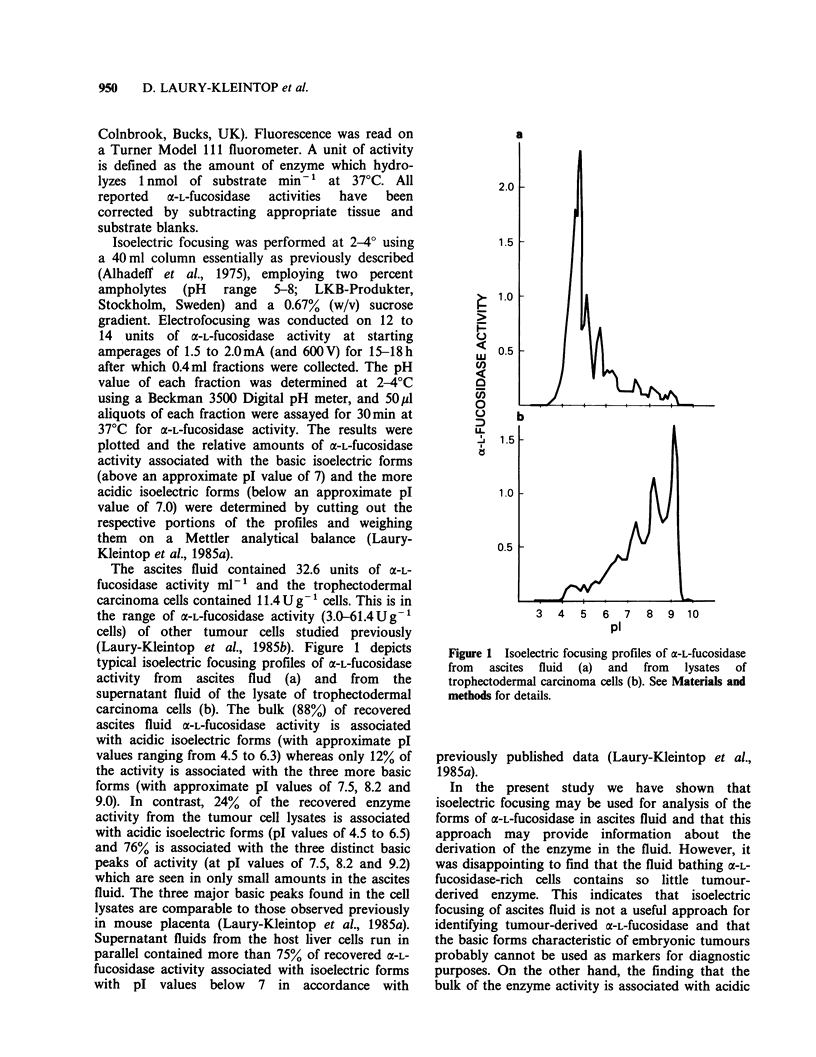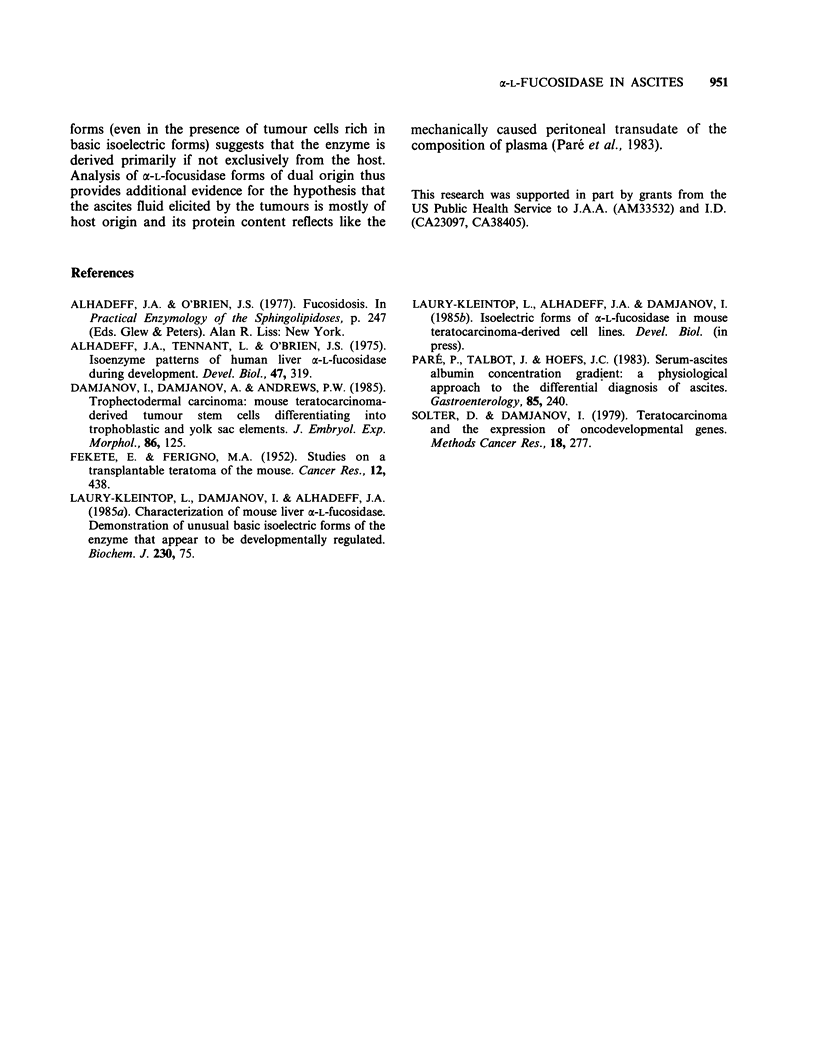# Ascites trophectodermal carcinoma cells exhibit embryonic mouse alpha-L-fucosidase isoenzyme pattern whereas the fluid exhibits adult mouse pattern.

**DOI:** 10.1038/bjc.1985.283

**Published:** 1985-12

**Authors:** L. D. Laury-Kleintop, J. A. Alhadeff, I. Damjanov


*Br. J. Cancer* (1985), **52**, 949–951

### Short Communication

### Ascites trophectodermal carcinoma cells exhibit embryonic mouse α-L-fucosidase isoenzyme pattern whereas the fluid exhibits adult mouse pattern

L.D. Laury-Kleintop[1], J.A. Alhadeff[1] & I. Damjanov[2]

[1]*Department of Chemistry, Lehigh University, Bethlehem, Pennsylvania 18015; and* [2]*Department of Pathology and Laboratory Medicine, Hahnemann University School of Medicine, Philadelphia, Pennsylvania 19102-1192, USA.*

α-L-fucosidase (α-L-fucoside fucohydrolase EC 3.2.1.51) is an enzyme that can be separated by isoelectric focusing into several isoelectric forms (Alhadeff & O'Brien, 1977). In contrast to most mammalian species which contain predominantly acidic and neutral forms of α-L-fucosidase, mouse tissues also contain unusual basic forms of the enzyme with approximate pI values of 8.3 and 9.0 (Laury-Kleintop *et al.*, 1985*a*). These basic forms are most prominent in the foetal tissues and placenta where they may account for up to 75% of the total activity. In contrast, adult tissues contain only 20–25% of basic forms. Embryonal carcinoma, tumours whose stem cells correspond to pluripotent embryonic cells from early stages of development (Solter & Damjanov, 1979), also contain, in accordance with their embryonic nature, predominantly the basic isoelectric forms of α-L-fucosidase (Laury-Kleintop *et al.*, 1985*b*).

Our studies of mouse α-L-fucosidase have so far concentrated on the enzyme from solid organs or homogeneous cell populations grown in culture (Laury-Kleintop *et al.*, 1985*a*, *b*). The present study was undertaken to determine whether the acidic and basic forms of α-L-fucosidase could also be found in body fluids. To that end we have analyzed ascites fluid, produced by injecting a trophectodermal carcinoma into the peritoneal cavity (Damjanov *et al.*, 1985). This tumour was chosen for two reasons: because it represents a malignant equivalent of trophectoderm and could thus (like placenta) be a good source of the embryonic (basic) forms of α-L-fucosidase; and because it grows readily in ascites form and causes rapid accumulation of ascites fluid. By studying the forms of α-L-fucosidase in the ascites fluid we hoped to determined whether the enzyme is derived from the adult host or released from the placenta-like tumour cells. We hypothesized that the predominance of embryonic (basic) forms would indicate that the enzyme in the fluid is mostly of tumour cell origin. On the other hand, if the tumour-induced accumulation of fluid is just a transudate of proteins produced by the host, then one would expect it to contain predominantly acidic isoforms of α-L-fucosidase.

Ascites was produced by injecting $1–2 \times 10^6$ trophectodermal carcinoma E6246D cells into adult outbred female Swiss-Webster mice. This tumour cell line, originally derived from serial transplants of a spontaneous ovarian teratocarcinoma of a C3H/Fe mouse (Fekete & Ferigno, 1952), was fully characterized and shown to be developmentally and immunochemically equivalent to trophectodermal cells in the preimplantation stage mouse embryos (Damjanov *et al.*, 1985). The tumour was allowed to grow for 7–10 days during which time it produced ~25–30 ml of clear ascites fluid. The animals were sacrificed by cervical dislocation and the fluid containing tumour cells was harvested into a glass container. Special care was taken to avoid contamination of the ascites fluid with blood, and all the animals with bloody ascites were discarded and not included into the study. Ascites was pooled from 3 animals and prepared for iso-electric focusing as follows. The cells were separated from the fluid by centrifugation at 12,000 *g* for 20 min. The supernatant was collected for further analysis and the cells were washed three times in 0.9% (w/v) NaCl to remove all traces of the ascites fluid. The washed cells were resuspended 1:3 (w/v) 10 mM, pH 5.0, citric acid–sodium citrate buffer containing 0.02% (w/v) $NaN_3$. The cells were lysed by five cycles of freeze-thawing in liquid nitrogen and the resulting homogenates were centrifuged at 12,000 *g* for 20 min. The supernatant fluids and resuspended pellets (after washing once with 10 mM, pH 5.0, citric acid–sodium citrate buffer) were assayed for α-L-fucosidase activity under conditions of linearity essentially as previously described (Alhadeff & O'Brien, 1977) at 37°C using 1.0 mM 4-methylumbelliferyl-α-L-fucopyranoside (Koch-Light Ltd.,

Correspondence: I. Damjanov.
Received 3 July 1985.

© The Macmillan Press Ltd., 1985

Colnbrook, Bucks, UK). Fluorescence was read on a Turner Model 111 fluorometer. A unit of activity is defined as the amount of enzyme which hydrolyzes 1 nmol of substrate $min^{-1}$ at 37°C. All reported α-L-fucosidase activities have been corrected by subtracting appropriate tissue and substrate blanks.

Isoelectric focusing was performed at 2–4° using a 40 ml column essentially as previously described (Alhadeff *et al.*, 1975), employing two percent ampholytes (pH range 5–8; LKB-Produkter, Stockholm, Sweden) and a 0.67% (w/v) sucrose gradient. Electrofocusing was conducted on 12 to 14 units of α-L-fucosidase activity at starting amperages of 1.5 to 2.0 mA (and 600 V) for 15–18 h after which 0.4 ml fractions were collected. The pH value of each fraction was determined at 2–4°C using a Beckman 3500 Digital pH meter, and 50 μl aliquots of each fraction were assayed for 30 min at 37°C for α-L-fucosidase activity. The results were plotted and the relative amounts of α-L-fucosidase activity associated with the basic isoelectric forms (above an approximate pI value of 7) and the more acidic isoelectric forms (below an approximate pI value of 7.0) were determined by cutting out the respective portions of the profiles and weighing them on a Mettler analytical balance (Laury-Kleintop *et al.*, 1985*a*).

The ascites fluid contained 32.6 units of α-L-fucosidase activity $ml^{-1}$ and the trophectodermal carcinoma cells contained 11.4 U $g^{-1}$ cells. This is in the range of α-L-fucosidase activity (3.0–61.4 U $g^{-1}$ cells) of other tumour cells studied previously (Laury-Kleintop *et al.*, 1985*b*). Figure 1 depicts typical isoelectric focusing profiles of α-L-fucosidase activity from ascites flud (a) and from the supernatant fluid of the lysate of trophectodermal carcinoma cells (b). The bulk (88%) of recovered ascites fluid α-L-fucosidase activity is associated with acidic isoelectric forms (with approximate pI values ranging from 4.5 to 6.3) whereas only 12% of the activity is associated with the three more basic forms (with approximate pI values of 7.5, 8.2 and 9.0). In contrast, 24% of the recovered enzyme activity from the tumour cell lysates is associated with acidic isoelectric forms (pI values of 4.5 to 6.5) and 76% is associated with the three distinct basic peaks of activity (at pI values of 7.5, 8.2 and 9.2) which are seen in only small amounts in the ascites fluid. The three major basic peaks found in the cell lysates are comparable to those observed previously in mouse placenta (Laury-Kleintop *et al.*, 1985*a*). Supernatant fluids from the host liver cells run in parallel contained more than 75% of recovered α-L-fucosidase activity associated with isoelectric forms with pI values below 7 in accordance with previously published data (Laury-Kleintop *et al.*, 1985*a*).

**Figure 1** Isoelectric focusing profiles of α-L-fucosidase from ascites fluid (a) and from lysates of trophectodermal carcinoma cells (b). See **Materials and methods** for details.

In the present study we have shown that isoelectric focusing may be used for analysis of the forms of α-L-fucosidase in ascites fluid and that this approach may provide information about the derivation of the enzyme in the fluid. However, it was disappointing to find that the fluid bathing α-L-fucosidase-rich cells contains so little tumour-derived enzyme. This indicates that isoelectric focusing of ascites fluid is not a useful approach for identifying tumour-derived α-L-fucosidase and that the basic forms characteristic of embryonic tumours probably cannot be used as markers for diagnostic purposes. On the other hand, the finding that the bulk of the enzyme activity is associated with acidic forms (even in the presence of tumour cells rich in basic isoelectric forms) suggests that the enzyme is derived primarily if not exclusively from the host. Analysis of α-L-focusidase forms of dual origin thus provides additional evidence for the hypothesis that the ascites fluid elicited by the tumours is mostly of host origin and its protein content reflects like the mechanically caused peritoneal transudate of the composition of plasma (Paré *et al.*, 1983).

This research was supported in part by grants from the US Public Health Service to J.A.A. (AM33532) and I.D. (CA23097, CA38405).

### References

ALHADEFF, J.A. & O'BRIEN, J.S. (1977). Fucosidosis. In *Practical Enzymology of the Sphingolipidoses*, p. 247 (Eds. Glew & Peters). Alan R. Liss: New York.

ALHADEFF, J.A., TENNANT, L. & O'BRIEN, J.S. (1975). Isoenzyme patterns of human liver α-L-fucosidase during development. *Devel. Biol.*, **47**, 319.

DAMJANOV, I., DAMJANOV, A. & ANDREWS, P.W. (1985). Trophectodermal carcinoma: mouse teratocarcinoma-derived tumour stem cells differentiating into trophoblastic and yolk sac elements. *J. Embryol. Exp. Morphol.*, **86**, 125.

FEKETE, E. & FERIGNO, M.A. (1952). Studies on a transplantable teratoma of the mouse. *Cancer Res.*, **12**, 438.

LAURY-KLEINTOP, L., DAMJANOV, I. & ALHADEFF, J.A. (1985*a*). Characterization of mouse liver α-L-fucosidase. Demonstration of unusual basic isoelectric forms of the enzyme that appear to be developmentally regulated. *Biochem. J.* **230**, 75.

LAURY-KLEINTOP, L., ALHADEFF, J.A. & DAMJANOV, I. (1985*b*). Isoelectric forms of α-L-fucosidase in mouse teratocarcinoma-derived cell lines. *Devel. Biol.* (in press).

PARÉ, P., TALBOT, J. & HOEFS, J.C. (1983). Serum-ascites albumin concentration gradient: a physiological approach to the differential diagnosis of ascites. *Gastroenterology*, **85**, 240.

SOLTER, D. & DAMJANOV, I. (1979). Teratocarcinoma and the expression of oncodevelopmental genes. *Methods Cancer Res.*, **18**, 277.